# Supplementary material for: Everyday Experiences of People Living with Mild Cognitive Impairment or Dementia: A Scoping Review
Source: Int J Environ Res Public Health. 2022 Aug 30;19(17):10828. doi: 10.3390/ijerph191710828 (PMC9518176; doi:10.3390/ijerph191710828)
Supplement: Supplementary file 1 [file ijerph-19-10828-s001.zip › ijerph-1870873-Supplementary.pdf]

Table S1. Characteristics of included studies.

Number	Author, year	Country	Focus of the study	Participants (population)	Method of data collection
1	Bartlett (2019) [66]	UK	How vulnerability is experienced and dealt with by people with dementia when outdoors	15 pwd; age: 55-85 years; gender: 5 female, 10 male	Walking interviews, sit-down interviews
2	Berg (2013) [67]	Sweden	Exploring experiences among individuals who have lived with MCI over seven years without converting to dementia	17 pwMCI; age: 57-86 years; gender: 6 female, 11 male	Semi-structured interviews
3	Borley (2016) [68]	UK	Exploring the meaning a woman with AD gives to receiving assistance with instrumental activities of daily living from her spouse	1 person with AD; age: 83 years; gender: 1 female	Semi-structured interviews
4	Brorsson (2011) [69]	Sweden	Experiences of accessibility in public space in people with AD	7 persons with AD; age: 63-80 years; gender: 5 female, 2 male	In-depth interviews
5	Buggins (2021) [70]	UK	How resilience is represented and lived in dementia	8 pwd (different types of dementia); age: 68-82 years; gender: 3 female, 5 male	Open interviews
6	Castaño (2019) [71]	Unknown	How metaphor is mobilized to describe the lived experience of dementia in illness narratives compiled from blogs by individuals with early-onset dementia	10 pwYOD; age not mentioned; gender: 5 female, 5 male	Analysis of blogs
7	Cedervall (2015) [72]	Sweden	How people with mild AD reason about physical activity as part of everyday life	14 persons with mild AD; age: 59-79 years; gender: 8 female, 6 male	In-depth interviews
8	Chen (2019) [73]	Taiwan	How elderly people with dementia in Taiwan view their lives given their cognitive impairments	12 persons with mild dementia; age: 78-94 years; gender: 6 female, 6 male	In-depth interviews
9	Chung (2019) [74]	UK	Experiences of people with dementia who had used the Homecare Enablement Services (HES)	16 pwd (different types of dementia); age: 70-90 years; gender: 8 female, 8 male	Semi-structured interviews
10	Clark (2020) [75]	UK, Sweden	How neighbourhoods support the well-being and everyday lives of people with dementia and their carers	67 pwd (different types of dementia); age: 51-88 years; gender: not mentioned	Hometours, walking interviews, network maps

11	Clemerson (2014) [76]	UK	Experience of living and coping with AD in younger life from the perspective of those diagnosed	8 persons with young onset Alzheimer; age: 35-63 years; gender: 2 female, 6 male	Semi-structured interviews
12	Dooley (2021) [77]	UK	Exploring post-diagnosis life with dementia	5 persons with recent diagnoses of dementia (max 2 years) (different types of dementia); age: 57-77 years; gender: 3 female, 2 male	Photovoice
13	Duane (2011) [78]	Australia	Understanding of the lives, strengths and capacities of older people who live alone with cognitive impairment or early-stage dementia	19 pwYOD; age: 69-93 years; gender: 13 female, 6 male; living alone	Interviews, fieldnotes
14	Frazer (2011) [79]	UK	Experiences of older women living alone, how they manage their identities and cope with day-to-day living	8 pwd (different types of dementia; 3 mild; 5 moderate/severe); age: 75-95 years; 8 females; living alone	Semi-structured interviews
15	Griffin (2016) [80]	UK	How people with behavioural-variant frontotemporal dementia make sense of their difficulties	5 people with bvFTD age: 46-62 years; gender: 2 female, 3 male; living with partner	Semi-structured interviews
16	Hedman (2016) [81]	Sweden	How persons with MCI relate to technology as a part of and as potential support in everyday life – both present and future	6 pwMCI; age: 61-86 years; gender: 2 female, 4 male	In-depth interviews, fieldnotes
17	Hellström (2015) [82]	Sweden	How older women with dementia express the importance of their homes and their chores in everyday life	7 pwd; age: 65-84 years; gender: female; married	Interviews, fieldnotes
18	Hicks (2021) [83]	UK	To examine the lived experiences of older men of rural life and its role in enabling and hindering their social inclusion	17 pwd; age: 68-90 years; gender: male	Open interviews, walking interviews, fieldnotes
19	Johannessen (2013) [84]	Norway	How people experience living with YOD	20 pwYOD; age: 54-67 years; gender: 8 female, 12 male	Interviews
20	Johannessen (2014) [85]	Norway	Interpreting metaphorical expressions of the lived experiences of everyday life in people with YOD	20 pwYOD; age: 54-67 years; gender: 8 female, 12 male (same	Secondary analysis of interview data

				participants as Johannessen et al., 2013)	
21	Johannessen (2019) [86]	Norway	How people living alone with YOD experience and cope with transitions during the progression of dementia	10 pwYOD (different types of dementia);<br>age: 49-67 years;<br>gender: 7 female, 3 male; living alone	Longitudinal: interviews
22	Johansson (2011) [87]	Sweden	Self-description of managing mealtime tasks by persons with dementia	15 pwd;<br>age: 69-86 years;<br>gender: 10 female, 5 male	Informal interviews, observations
23	Johansson (2015) [88]	Sweden	Experiences of cognitive impairment, its consequences in everyday life and need for support in people with MCI or mild dementia	5 pwMCI, 8 persons with mild dementia;<br>age: 58-78 years;<br>gender: 6 female, 7 male	Interviews
24	Lin (2021) [89]	Hongkong	Experience of neuropsychiatric symptoms (NPS) among females with MCI	29 pwMCI;<br>age: 55-88 years;<br>gender: 29 female	Semi-structured telephone interviews
25	Lloyd (2015) [90]	Australia	Understanding of the everyday lives and unmet service needs of people with dementia who live alone	7 persons with early to moderate dementia;<br>age: 48-85 years;<br>gender: not mentioned; living alone	Semi-structured in-depth interviews
26	Margot-Cattin (2021) [91]	Switzerland	How familiarity is experienced by persons with dementia performing activities and visiting places outside home and how familiarity might contribute to maintaining participation	9 pwd (various stages);<br>age: 65-90 years;<br>gender: 4 female, 5 male	Home based interviews, walking interviews
27	Mazaheri (2013) [92]	Iran	People's experiences of living with dementia in Iran	15 pwd (AD or vascular);<br>age: 60-87 years;<br>gender: 6 female, 9 male	Semi-structured interviews
28	McDuff (2015) [93]	Canada	The meaning of activity in the everyday lives of people living with dementia	12 persons with recent diagnoses (0-3 years; mild to moderate stage;<br>age: 65-86 years;<br>gender: 6 female, 6 male	Conversational style interviews

29	Mitchell (2020) [94]	UK	Current public perceptions of dementia along with the facilitators and barriers to living well from the perspective of people living with the condition in Northern Ireland	20 persons with early to middle stage of dementia (different types of dementia); age: 52-78 years; gender: 14 female, 6 male	Focus groups
30	Moe (2021) [95]	Norway	The factors that influence everyday coping strategies as described by persons with early to intermediate dementia	12 persons with early to moderate stage of dementia; age: 62-86 years; gender: 3 female, 9 male	Semi-structured interviews
31	Odzakovic (2020) [96]	Sweden	The experience of the neighbourhood for people with dementia, to understand the meaning that neighbourhood held for the participants	14 pwd; age: 62-87 years; gender: 3 female, 11 male (same participants as in Clark et al., 2020)	Walking interviews
32	Odzakovic (2021) [97]	UK, Sweden	Experiences of people with dementia who live alone, how they establish social networks and relationships in a neighbourhood context, and how they are supported to maintain this social context within everyday life	14 pwd (different types of dementia); age: 62-88 years; gender: 11 female, 3 male (same participants as in Clark et al., 2020)	Walking and semi-structured interviews, home-tours, social network mapping
33	Parikh (2016) [98]	Canada	How memory changes impact everyday lives	14 persons with anamnestic MCI; age: 73-89 years; gender: 4 female, 10 male	Focus groups
34	Pipon-Young (2011) [99]	UK	The experiences of younger people with dementia, the support that has been beneficial, to identify areas in need of change and to draw out the key problem areas	8 pwYOD (7 persons with AD, 1 mixed dementia); age: 60-67 years; gender: 7 female, 1 male	Semi-structured interviews, action research groups
35	Portacolone (2018) [100]	USA	The overall experience of older adults living alone with cognitive impairment in order to better understand their priorities, needs, and concerns	12 persons with MCI or AD; age: 71-85 years; gender: 10 female, 2 male; living alone	Open-ended interviews, participant observation
36	Rabanal (2018) [101]	UK	The experiences and needs of people living with YOD to gain an understanding of the issues that impact on them	14 pwYOD; age: 57-67 years; gender: not mentioned	Semi-structured interviews
37	Renn (2021) [102]	USA	The subjective experience of a typical week living with MCI: the important daily activities, barriers to usual activities, and facilitators and supports	11 pwMCI; age: 57-79 years; gender: 5 female, 6 male	Photo elicitation semi-structured interviews
38	Roberts (2013) [103]	UK	The meta representational level of awareness in relation to the experience of living with MCI, and particularly the psychological impact of living	25 pwMCI; age: 60-97 years; gender: 9 female, 16 male	Semi-structured interviews

			with memory difficulties and how these impact on daily life.		
39	Robertson (2014) [104]	UK	How self and social identity are represented in an individual's narrative of the quality of everyday life	1 pwd; age: >80 years; gender: female; living alone	Conversational style interviews
40	Robinson (2012) [105]	Sweden	How one person experienced the early years of dementia as she was living through the pre-clinical and early clinical stages of AD	1 person with early-stage AD; age: 64 years; gender: female	Longitudinal: interviews
41	Rostad (2013) [106]	Norway	Experience of people with YOD, and the meanings that might be found in those experiences	4 pwYOD (3 with AD, 1 with vascular); age: 55-62 years; gender: 2 female, 2 male	Narrative interviews
42	Sakamoto (2017) [107]	Canada	The perception of personhood in younger adults with dementia	4 pwYOD in early stages (different types of dementia); age: <65 years; gender: male	Interviews, art-based data
43	Sandberg (2017) [108]	Sweden	How persons with dementia, living at home, experience risks in their daily life and how they handle these situations	12 persons with mild to moderate dementia (different types of dementia); age: 67-87 years; gender: 6 female, 6 male	Open-ended interviews
44	Steeman (2013) [109]	Belgium	Exploring changes in the experience of living with dementia	17 persons with early-stage dementia (different types of dementia); age: 72-91 years; gender: 15 female, 2 male	Longitudinal: open interviews
45	Steenwinkel (2014) [110]	Belgium	An in-depth understanding of person–space relationships	1 person with early onset AD; age: <60; gender: female	Semi-structured interviews
46	Strandenæs (2017) [111]	Norway	Experiences with day care designed for people with dementia	17 persons with mild or moderate dementia; age: 72-92 years; gender: 10 female, 7 male	Qualitative inquiry: semi-structured interviews
47	Sturge (2020) [112]	the Netherlands	Using the concept of activity space to examine the social health of older adults with memory problems and dementia who live at home	7 pwd; age: 59-93 years; gender: 5 female, 2 male	Walking and in-depth interviews, GPS data, travel diary entries

48	Sturge (2021) [113]	the Netherlands	Identifying assets that contribute to the well-being of people with memory problems and dementia living in a community context in the Netherlands	8 pwd;<br>age: 59-93 years;<br>gender:<br>6 female, 2 male (same participants as Sturge et al., 2020)	Sociodemographic survey, GPS data, diaries, walking and in-depth interviews
49	Svanström (2015) [114]	Sweden	Elucidating the phenomenon of living alone with dementia and having a manifest care need.	6 pwd;<br>age: 80-90 years;<br>gender: 5 female; 1 male; living alone	Fieldnotes, conversations
50	Talbot (2021) [115]	UK	Why people with dementia use Twitter and what challenges they face when using Twitter	11 pwYOD (different types of dementia);<br>age: 48-66 years;<br>gender: 3 female, 8 male	Semi-structured interviews
51	Thoft (2020) [116]	Denmark	The lifeworld perspective of how people with mild dementia experience and manage everyday life with dementia.	12 persons with mild dementia (different types of dementia);<br>age: 65-79 years;<br>gender: 3 female, 9 male	In-depth interviews
52	Thorsen (2020) [117]	Norway	The experience of the quality of life with YOD as a single person.	1 pwYOD, shortly after diagnosis;<br>age: around 50 years;<br>gender: female	In-depth interviews, open dialogue, over a period of 3 years
53	Trindade (2018) [118]	Brazil	Assessing different objects of awareness of disease of people with AD in mild and moderate stages	34 persons with mild and moderate AD;<br>age: mean 71.84;<br>gender: 19 female, 15 male	Semi-structured interviews
54	Trindade (2020) [119]	Brazil	The experience of awareness of functional activities in people with mild and moderate AD	38 people with mild and moderate AD;<br>age: mild mean 70, moderate mean 73;<br>gender: 24 female, 14 male	Semi-structured interviews
55	Vliet (2017) [120]	the Netherlands	Exploring the aspects of daily life that give people with YOD a sense of usefulness	18 pwYOD (different types of dementia, different stages);<br>age: mean 63.5;<br>gender: 7 female, 11 male	Focus groups
56	Ward (2020) [121]	Denmark	Evaluating the service provided by an adult school by understanding people with dementia’s experiences of being a student.	10 pwd (different types of dementia);<br>age: 67-83 years;<br>gender: 5 female, 5 male	Photo elicitation and storytelling with visual methods in groups

57	Wijngaarden (2019) [122]	the Netherlands	Day-to-day experiences by providing an idiographic description of what it means existentially to be in the world as a person with dementia	part 1: 16 pwd; age: 40-89 years; gender: 8 female, 8 male<br>part 2: 37 pwd; age: 40-99; different types of dementia<br>gender: 20 female, 17 male	Part 1: diaries, part 2: narrative and semi-structured interviews
58	Xanthopoulou (2019) [123]	UK	People's experiences of cognitive decline and receiving a diagnosis of dementia soon after receiving the diagnosis	61 persons with mild or moderate dementia (<2 years after diagnosis, different types of dementia);<br>age: 65-91 years;<br>gender: 34 female, 27 male	Semi-structured interviews

Notes: AD: Alzheimer's Disease. bvFTD: behavioural Frontotemporal Disease. MMSE: Mini-Mental State Examination. pwd: people with dementia. pwMCI: people with MCI. pwYOD: people with young-onset dementia. YOD: young-onset dementia. UK: United Kingdom. USA: United States of America.
